# The Mosquito Repellent Activity of the Active Component of Air Freshener Gel from Java Citronella Oil (*Cymbopogon winterianus*)

**DOI:** 10.1155/2020/9053741

**Published:** 2020-01-29

**Authors:** Willy Tirza Eden, Dante Alighiri, Kasmadi Imam Supardi, Edy Cahyono

**Affiliations:** ^1^Essential Oil Study Center, Faculty of Mathematics and Natural Sciences, Universitas Negeri Semarang, 50229 Central Java, Indonesia; ^2^Chemistry Department, Faculty of Mathematics and Natural Sciences, Universitas Negeri Semarang, 50229 Central Java, Indonesia

## Abstract

This study examines the active component of *Cymbopogon winterianus* (Java citronella) oil, as a green mosquito repellent, obtained through a steam distillation method. Java citronella oil, which contains citronellol, citronellal, and geraniol, was isolated by batch vacuum fractional distillation, and their effect was tested against the dengue fever (DF) vector, known as *Aedes aegypti*. Furthermore, air freshener gels were formulated with Java citronella oil, carrageenan, gum, sodium benzoate, ethylene glycol, polysorbate 20, sodium chloride, and distilled water, at varying concentrations. The results show that formula I has the best controlled release evaporation for citronellal, citronellol, and geraniol, as well as the best storage time of 16.82 days and 12.77 days for geraniol and citronellol, respectively. The most significant specific gravity (0.0136) was recorded in formula V, while gel formula I exhibited the highest level of instability at 35°C, with a syneresis value of 77.11% in *t* = 72 h and pH 5.33. In addition, formula IV at 5°C demonstrated the highest syneresis (75.34%) in *t* = 72 h, with pH 7.04, while a peak viscosity of 100,958 cP was recorded in formula IV. The repellent activity of each active component was measured based on the period of protection conferred against the bites of *Aedes aegypti* within one hour, and the results showed geraniol and citronellol, with respective activity of 78.00% ± 4.83 and 77.34% ± 3.57, as the most effective.

## 1. Introduction

Dengue fever (DF) is a mosquito-borne viral disease, known to be one of the major life-threatening health problems, in the absence of proper vaccine or treatment. Annually, an estimate of 390 million infections occur worldwide [1], and data from 76 countries show a double fold increase every decade between 1990 and 2013, with the highest cases being reported in Asia [2]. Moreover, dengue virus is transmitted by female mosquitoes mainly of the species *Aedes aegypti*.

The most prominent approaches adopted in the vector control are based on the use of chemical insecticides containing DEET (*N,N-diethyl-3-methyl benzamide*), known to permeate the skin, and also cause allergic and toxic reactions on application, with odor that is unpleasant to some people [3, 4]. In addition, essential oils from aromatic plants have been suggested as alternative sources of mosquito repellent, particularly those from the Citronella genus (Poaceae), commonly adopted as ingredients [5].

*Cymbopogon winterianus*, commonly known as Java citronella, is native to the tropical and subtropical areas of Asia, India, and Indonesia, consisting of citronellol, citronellal, and geraniol as its major compounds [6]. Java citronella oil has been registered in the US EPA (United States Environmental Protection Agency) as insect repellent due to its efficacy and low toxicity. It has been proven to exhibit low acute toxicity in laboratory animals [7]. Furthermore, the oil extract has been adopted as an antiseptic, antispasmodic, diuretic, and febrifuge [8], despite its several limitations observed. This includes the characteristics of short-lived effectiveness, occurring as a result of the active ingredients' high volatility [9]. Various studies have evaluated the mosquito repellent activity of citronella oil extracted from *Cymbopogon* genus, particularly *C. nardus*, while *C. winterianus* and its active components have not been extensively investigated. This study, therefore, assesses the mosquito repellency of the active components obtained from *C. winterianus* essential oil, encompassing citronellal, citronellol, and geraniol, against *Aedes aegypti.* In addition, its formula optimization in the form of an air freshener gel was also conducted to improve the essential oil longevity.

## 2. Materials and Methods

### 2.1. Essential Oils and Chemicals

*Cymbopogon winterianus* herbs were collected from Boyolali District (7°31′59^″^S/110°35′44^″^E), Central Java, Indonesia, dried for one day, and the essential oil was subsequently obtained through a steam distillation method [10]; its active ingredients were isolated using fractional vacuum distillation and GC–MS. GC–MS analysis was carried out on an Agilent Technologies GC–MS instrument equipped with a GC 7890A gas chromatograph and an MS 5975C VL MSD mass spectrometer detector and provided with an HP-5MS capillary column. The data acquisition and data processing were performed using the MSD Chemstation E.01.01.335 (Agilent) software. Also, all other chemicals required for formulating the air freshener gel were purchased from Sigma-Aldrich and of reagent grade.

### 2.2. Mosquito Repellent Activity Test

The *Aedes aegypti* colony was reared in the Insecticide Assessment Laboratory of the Research and Development Center for Vector Health and Disease Reservoirs. The repellence of citronellal, citronellol, and geraniol was evaluated against the *Aedes aegypti* females presented in cages and tested under laboratory conditions as described by Kim et al., where method description partly reproduces wording [3]. The cage was divided into three compartments, while a rat was restricted in a wire net, and then the tested samples (1 g) were placed in the first compartment (20 × 20 × 20 cm) of polyester netting (200 mesh) woven with stainless frames. In addition, a disposable piece of cardboard with a center hole (9 cm) was inserted into one-third of the second square section (acrylic cage, 20 × 20 × 60 cm length), while the first and third polyester nettings were fitted at each end of this acrylic cage. Conversely, the third compartment was designed identically with the first, to which 30 nonblood-fed female mosquitoes (aged 5-10 d) were introduced, and the number that migrated into the treated compartment was counted at 5, 10, 15, 30, 45, and 60 minutes postapplication. In addition, all tests were performed five times, and mosquito repellency was calculated using the following equation:

(1) $$\begin{array}{c} \%\text{repellency}=\frac{C-T}{C}\times 100\%, \end{array}$$

where *C* indicates the number released and *T* was the amount identified in the cage, the chamber containing a mouse, and the volunteer.

### 2.3. Statistical Analysis

The data obtained were compared using *F*-tests, and a *P* value < 0.05 was considered statistically significant, using SPSS as the analytical tool.

### 2.4. Ethics

The study protocol was approved by the Health Research Ethics Committee of Universitas Negeri Semarang, Indonesia.

### 2.5. Formulation of Air Freshener Gel

The process of optimizing the formula for air freshener gel with 1% Java citronella oil was conducted using various concentrations of the relevant ingredients, as shown in Table 1.

**Figure 1:**
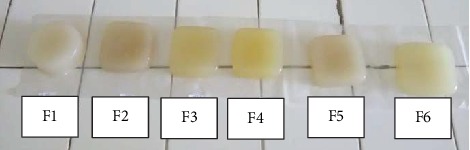
Six formulations of air freshener gel.

Distilled water was added to carrageenan, sodium benzoate, and NaCl, which was then stirred, followed by heating of the mixture at a temperature of 75°C, which was then lowered to 65°C. In addition, the gum, ethylene glycol, and polysorbate 20 were incorporated and stirred until dissolved; 1% of Java citronella oil was added to the mixture after removal from heat. This formulation was stirred to achieve homogeneity and then subsequently inserted in the mold and allowed to stand at room temperature [11]. The samples are demonstrated in Figure 1.

### 2.6. Control Release and Storage Life

The six formulations of air freshener gel were incubated in room temperature, and a small amount was collected at a certain time (*t* = 5, *t* = 10, *t* = 15, *t* = 30, *t* = 45, and *t* = 60). These were tested using GC-MS, and the remaining component was measured to assess the concentration of citronellal, citronellol, and geraniol.

### 2.7. Physicochemical Test

Specific gravity of the gel was evaluated using a pycnometer (Iwaki) at room temperature, and the viscosity was determined with the cone and plate geometry viscometer (Brookfield Viscometer), characterized by a typical run of 1.5 rpm at 25°C, using spindle No. 64. Also, the formulations for pH measurement were prepared by dissolving 1 g of gel in 10 ml distilled water, which was further tested with a calibrated pH meter at 5°C and 35°C.

### 2.8. Syneresis Rate

Syneresis is the expulsion of liquid from a gel, which was evaluated by storing the samples at temperatures 5°C and 35°C for 24, 48, and 72 h. These were subsequently placed on a dish to collect the water released during the storage time, and syneresis rate was calculated by determining and comparing the weight loss through the procedure with the initial value.

## 3. Result and Discussion

### 3.1. Mosquito Repellency of Citronellal, Citronellol, and Geraniol

There has been an upsurge in the use of plant-based mosquito repellents, due to the lack of adverse effects on humans, as against the synthetic repellents, including DEET (*N,N-diethyl-3-methyl benzamide*). Furthermore, essential oil obtained from *Cymbopogon* genus is one of the most commercially available products in the market, and previous reports suggest repellent activity against *Aedes albopictus*, which tends to decrease over time, from 97.9% at 0 h to 71.4% at 1 h and 57.7% at 2 h [10]. Moreover, citronella oil from *C. excavatus* demonstrated 100% activity for 2 h against *Anopheles arabiensis* [12], and a mixture of *C. winterianus* oil and 5% vanillin provided 100% protection for 6 h against *Aedes aegypti*, *Culex quinquefasciatus*, and *Anopheles dirus*, compared favorably with 25% DEET [13].

*C. winterianus* (Java citronella) is native to Indonesia with abundant availability and known to contain citronellal, citronellol, and geraniol as its major components. These were all isolated using fractional vacuum distillation and identified using GC-MS. Citronellal, citronellol, and geraniol were identified at 13.6 min, 16.6 min, and 18.2 min, respectively. The mosquito repellency of three components against the DF's vector, *Aedes aegypti*, was assessed, and the results are provided in Table 2.

The repellent activity of each was measured by evaluating the protection period against the bites of *Aedes aegypti* within one hour. The result identified geraniol as the component with the highest activity (*t* = 5 min up to *t* = 60 min). The repellency estimated within a 60-minute period was 78.00% ± 4.83 for geraniol, 77.34% ± 3.57 for citronellol, and 71.33% ± 4.95 for citronellal. Despite the fact that statistical analysis showed no significant difference between citronellol and geraniol, the repellency of the latter substantially varied from the other two components, although all three reduced in effectivity over time as a result of their volatility. This result corresponds with previous reports that also demonstrate a decline in the rate of repellency for citronella oil [11, 14]; hence, the current study focuses on the production of an air freshener gel, as a strategy to control component release, decrease evaporation rate, and prolong the effects [15]. These gels possibly dampen the bad smells inside a room, causing an elevated feeling of comfort. Air freshener gel, which acts as mosquito repellent is still rarely found on the market. Air freshener gel product with carrageenan was found in US patent number 9.352.060 B2. In this patent, the air freshener formulation has a composition of perfume oils, dibutyl lauroyl glutamide, polyethylene glycol 1000, and carrageenan gel. Hence, in this study, we used carrageenan along with other materials to control the release of Java citronella oil's major compounds [16].

### 3.2. Controlled Release and Storage Life

The essential oil components tend to go through chemical changes on storage and handling, which ensues as a result of high volatility and decomposition caused by direct exposure to heat, humidity, light, or oxygen [17]. Therefore, the gel formulation is expected to help decrease volatility and subsequently control the component release and prolong the repellency effects, with release profile shown in Table 3.

From the *R*-square data, the citronellal, citronellol, and geraniol content in formula I was shown to have released steadily, in contrast with the others. Furthermore, geraniol and citronellol demonstrate a steady release with *R*‐square = 0.9699 and 0.9323, respectively, as well as the longest shelf life, at 16.82 and 12.77 days, respectively. Conversely, formula II demonstrated the best-controlled release action with the evaporation of citronellal, at an *R*-square value of 0.9993, while formula III had the best storage time of 22.40 days with citronellal.

### 3.3. Physicochemical Properties and Syneresis Rate of Air Freshener Gel

The air freshener formula was investigated for the following physicochemical properties, encompassing specific gravity, viscosity, pH, and syneresis. These were closely related with product stability, and the results are shown in Tables 4 and 5. Furthermore, the gelling agent known as carrageenan was used in this formulation as a natural polymer derived from carbohydrate. This has been widely adopted in the preparation of food and pharmaceuticals for gelling, thickening, emulsifying, and syneresis control, as well as for bodying, binding, and dispersion [18, 19]. Moreover, the largest specific gravity was observed in formula V, being 0.0135 (Table 4), while the specific gravity of formulas II and IV was not significantly different, according to the statistical analysis.

Carrageenan typically forms highly viscous aqueous solutions, with formulas I, II, and IV containing the highest concentrations at 3%, 2.5%, and 2.5%, respectively. This has, therefore, been associated with the gel viscosity, as the highest value obtained at a speed of 1.5 rpm (spindles 64) ensued in formula IV, at an average of 97930 cP. Moreover, the air freshener gel was formulated with a combination of carrageenan, NaCl as the salt component, and gum as a dispersing, emulsifying, and suspending agent. In addition, the gum possesses a high viscosity at lower temperatures, as well as pseudoplasticity, and nonsensitivity to temperature, pH, and electrolyte concentration [20]. This was observed in formula II, which has the highest amount, known to display nonsensitivity to pH, as the values recorded at 5°C and 35°C were not significantly different.

Syneresis is regarded as an instability phenomenon and also described as the “spontaneous contraction of a gel, accompanied by the expulsion of liquid from the pores” [21]. This has been reported in some protein and polysaccharide gels, including carrageenan [22]. In addition, formula IV 5°C exhibited the highest syneresis and pH of 75.34% in 72 h and 7.35, respectively, suggesting the prominent instability under low temperatures. Furthermore, the highest and most significantly different pH was attained in formula IV, while formula I exhibited a comparably higher instability at 35°C, with a syneresis of 77.11% in 72 h, with a pH value of 5.47. However, formula VI showed the least rate amongst all, at 5°C and 35°C.

Carrageenan contains 20-35% sulphate, which affects its viscosity, stability, and syneresis properties, and the results affiliated this effect to the high concentration and low pH, as indicated by the amount of water expelled. Based on the release and shelf life of citronella oil components, formula I was attributed as the most prospective, although the formula requires further optimization with regard to stability in high temperature.

## Figures and Tables

**Table 1:** Several formulas of air freshener gel with 1% Java citronella oil.

Component	Formula percentage (%)					
	I	II	III	IV	V	VI
Carrageenan	3	2.5	1.5	2.5	2	1.5
Gum	0.5	1	0.5	0.5	0.5	0.5
Sodium benzoate	0.3	0.3	0.3	1	1	1
Ethylene glycol	1	1	1	1.5	2	2
Polysorbate 20	0.2	0.2	0.5	0.5	0.5	1
NaCl	1	1	1	1	1	1
Aquadest	94	95	96	93	93	92

**Table 2:** The mosquito repellent activity of three major compounds isolated from *C. winterianus* oil.

Active component	Minute after treatment (% repellency activity, mean)					
	5	10	15	30	45	60
Citronellal	84.00	82.00	80.00	77.33	74.00	71.33
Citronellol	86.67	84.67	82.67	80.67	78.67	77.34
Geraniol	90.67	88.00	84.67	82.00	79.33	78.00

**Table 3:** Controlled release of citronellal, citronellol, and geraniol from air freshener gel formulations.

Formula	Citronellal		Citronellol		Geraniol	
	*R*-square	Shelf life (day)	*R*-square	Shelf life (day)	*R*-square	Shelf life (day)
I	0.8824	1.36	0.9323	12.77	0.9699	16.82
II	0.9993	15.06	0.4808	-*^a^*	0.4808	-*^a^*
III	0.9891	22.40	0.9695	1.62	0.3952	-*^a^*
IV	0.7500	-*^a^*	0.4696	-*^a^*	0.5057	-*^a^*
V	0.3952	-*^a^*	0.9845	1.12	0.4606	-*^a^*
VI	0.3952	-*^a^*	0.9308	4.04	0.8834	11.33

*^a^*Not determined.

**Table 4:** Physicochemical properties of air freshener gel formulations.

Formula	Specific gravity	Viscosity (cP)	pH after temperature	
			5°C	35°C
I	0.0016	34369	5.08	5.47
II	0.0052	47125	5.08	5.50
III	0.0044	37419	5.91	6.43
IV	0.0051	97930	7.35	6.35
V	0.0135	54425	5.23	5.55
VI	0.0063	35929	6.00	6.69

**Table 5:** Syneresis rate of air freshener gel formulation during storage time (24-72 h).

Formula	% syneresis rate after storage					
	5°C			35°C		
	24 h	48 h	72 h	24 h	48 h	72 h
I	29.88	53.81	71.29	26.31	50.69	77.11
II	25.10	39.75	57.73	27.03	50.89	69.32
III	29.12	44.60	55.64	30.66	52.05	71.29
IV	42.99	65.50	75.34	28.06	46.80	69.66
V	34.44	57.64	69.93	33.01	50.50	69.15
VI	5.03	10.15	16.70	26.14	43.70	62.73

## Data Availability

The mosquito repellency data, controlled release data, and all physicochemical investigation data used to support the findings of this study are available from the corresponding author upon request.
